# Operating room architecture is not a risk factor for surgical site infections

**DOI:** 10.1038/s41598-021-90574-z

**Published:** 2021-06-28

**Authors:** Thorsten Jentzsch, Lucas Kutschke, Patrick O. Zingg, Mazda Farshad

**Affiliations:** Department of Orthopaedics, Balgrist University Hospital, University of Zurich, Forchstrasse 340, 8008 Zurich, Switzerland

**Keywords:** Diseases, Health care, Medical research, Risk factors

## Abstract

Surgical site infection (SSI) may cause a substantial burden for patients and healthcare systems. A potential risk of different architectures of the operating room for SSI is yet unknown and was subject of this study. This observational cohort study was performed in a university hospital and evaluated patients, who underwent a broad spectrum of orthopedic surgeries in 2016 (open-plan operating room architecture) versus (vs) 2017 (closed-plan operating room architecture). Patients, who underwent surgery in the transition time period from the open-plan to the closed-plan operating room architecture and those, who were treated e.g. for osteomyelitis as index procedure were excluded. The primary outcome was revision surgery for early SSI within 30 (superficial) or 90 (deep or organ/space) days of surgery. Age, gender, American society of anesthesiologists (ASA) classification, and the body mass index (BMI) were considered as potential interacting factors in a logistic regression analysis. The incidence of revisions for SSI was 0.6 percent (%) (n = 45) in the 7'740 included surgical cases (mean age of 52 (standard deviation (SD) 19) years; n = 3'835 (50%) females). There was no difference in incidences of revision for SSI in the open- vs closed-plan operating room architecture (0.5% vs 0.7%; adjusted odds ratio (OR) = 1.34 (95% confidence interval (CI) 0.72–2.49, *P* = 0.35)). Age and gender were not a risk factor for revision for SSI. However, ASA classification and BMI were identified as risk factors for the incidence of revision for SSI (OR = 1.92 (95% CI 1.16- 3.18, *P* = 0.01) and OR = 1.05 (95% CI 1.00–1.11, *P* = 0.05)). The overall incidence of revisions for early SSI after a broad spectrum of orthopedic surgeries was relatively low (0.6%) and independent from the operating room architecture. An increase in ASA classification and possibly BMI, however, were identified as independent risk factors for revision for SSI.

## Introduction

Surgical site infections have an incidence of around 3% (1–7%) after orthopedic surgery^1^, mostly (~ 20%) caused by the pathogen Staphylococcus aureus^2^. They can be classified according to anatomical region, i.e. superficial (epidermis, dermis, and subcutaneous tissue), deep (fascia and muscle tissue), and organ/space^3^. One of the criteria for their diagnosis defines their occurrence within 30 days of a surgery if they are superficial or within 90 days if they are deep or organ/space-related (e.g. hip arthroplasty)^4^. They represent a complication that can cause significant burden for patients and the healthcare system. They increase the morbidity (e.g. sepsis), mortality (~ 3%), and economic burden (infections cost around 230 million per year in Switzerland)^5^. Therefore, risk factors for surgical site infection need to be identified and minimized.

Identified risk factors are categorized into modifiable (e.g. potentially obesity) and non-modifiable (e.g. demographic characteristics). Although entirely unknown so far^6^, a modifiable potential risk factor could be the operating room architecture (operating room architecture), namely open versus closed-plan operating room architecture. On the one hand, open-plan design likely has more operating room traffic by personnel, which could potentially increase the risk of infection^7^. On the other hand, it also offers more space to stay away from the sterile, laminar flow area, which could reduce the risk of infection^8^. Operating rooms play a crucial role in modern hospitals with around 50 million operations per year in the United States generating around half of the total hospital income^9,10^. For example, it can be important to know whether surgical site infection is influenced by operating room architecture for renovation of current and planning of future hospitals.

The objective of this study was to evaluate if the operating room architecture is a risk factor for surgical site infection. Our hypothesis was that the incidence of surgical site infection is higher in open-plan than in closed-plan operating room architecture.

## Materials and methods

This observational cohort study was performed in a single-center university hospital. It included patients, who underwent a broad spectrum of orthopedic surgeries between 2016–2017. The local ethics committee (Kantonale Ethikkomission Zurich) issued a waiver (BASEC Nr. Req-2018–00,521) for this study and informed consent due to the retrospective nature of the study anonymous data handling. All methods were in accordance with relevant guidelines and regulations.

**Figure 1:**
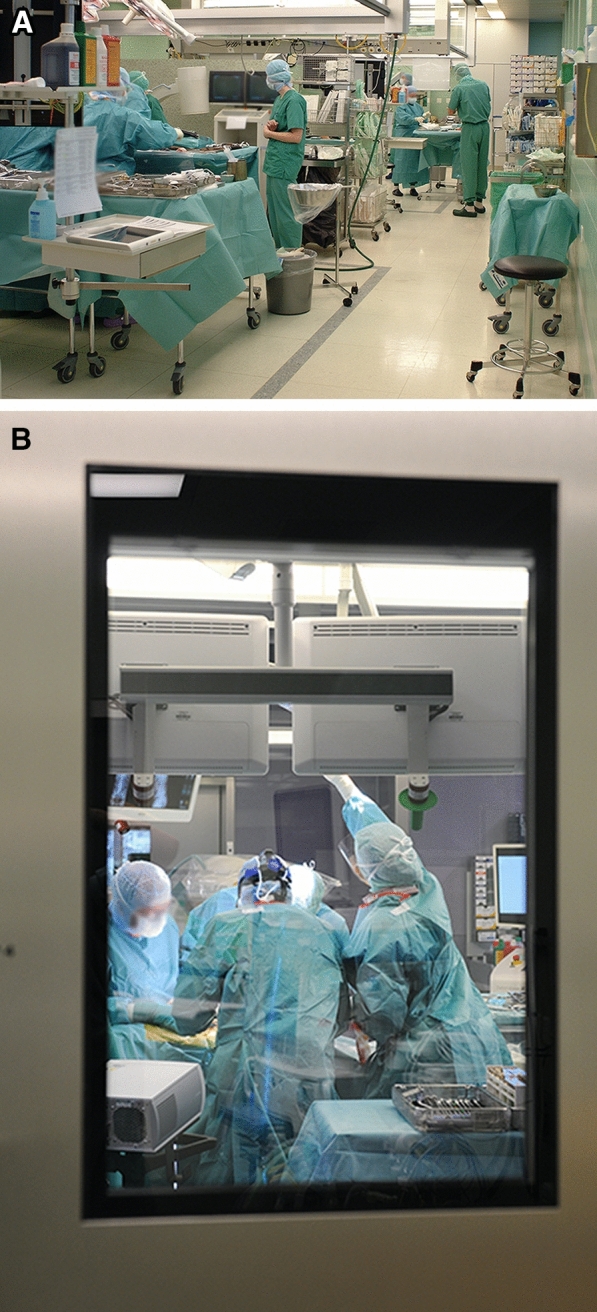
(**A**) Operating room architectures. The authors would like to acknowledge and thank Christian Streng for providing this picture and his permission to publish it. (**A**) Open-plan. The open-plan operating room architecture was used until 2016 and allowed simultaneous (up to four) surgeries within one large room separated by mobile dividers. (**B**) Closed-plan. The closed-plan operating room architecture has been used since 2017 and has allowed one surgery within one stand-alone room separated by actual walls.

The main exposure variable was the operating room architecture (open- versus (vs) closed-plan). Until 2016, the open-plan operating room architecture was used and allowed simultaneous (up to four) surgeries within one large room separated by mobile dividers (Fig. 1A). During this time, there was also an additional fifth operating room, which used a closed-plan operating room architecture and was mostly used for hand surgery. Since 2017, the closed-plan operating room architecture has been used in six operating rooms and has allowed only one surgery within each stand-alone room separated by actual walls and doors (Fig. 1B). Although the preoperative antibiotic regime was changed from cefuroxime 1.5 g to dosage adjusted to BMI after the first quarter of 2017, the main parts of the surgical routine (e.g. desinfection technique, manner of surgical field preparation, gloves, irrigation, routing sterilisation, and laminar flow) remained unchanged. Patients were excluded if they underwent surgery in the closed-plan OR in 2016 (n = 981), in the transition time period from the open-plan to the closed-plan operating room architecture (n = 72), underwent hand surgery (to exclude bias because most were performed in the separated closed-plan room in 2016) or primary septic surgery (e.g. for osteomyelitis) (n = 328). Age^11^, gender, American society of anesthesiologists (ASA) classification^12^, and the body mass index (BMI)^13^ were also evaluated as surrogates for the most important potentially interfering risk factors for surgical site infection.

The primary outcome was surgical revision due to early surgical site infection^3,4^. A surgical site infection was defined as present if the following criteria were met as suggested by the Centers for Disease Control and Prevention (CDC)^14^: A + B or A + C or A + B + C. The criterion A was defined as an infection within 30 days of surgery (superficial surgical site infection) or three months if an implant was present (deep or organ/space surgical site infection). Criterion B consisted of (1) pus, positive culture, pain/swelling/redness/erythema + surgical revision (but not if culture negative) for superficial surgical site infection, (2) pus, spontaneous dehiscence or surgical revision +  > 38 degree Celsius or pain (but not if culture negative), and abscess or frank infection at revision/histology/radiology for deep surgical site infection, and (3) pus, positive culture, and abscess or frank infection at revision/histology/radiology for deep or organ/space surgical site infections. Criterion C was a diagnosis of infection by a physician. The criteria for an arthroplasty differed to some extent. One of three criteria needed to be present. Criterion 1 consisted of two positive cultures. Criterion 2 included a fistula. Criterion 3 consisted of minor criteria (C-reactive protein > 100 mg per liter or erythrocyte sedimentation rate > 30 mm per hour, joint aspiration with > 10′000 leukocytes/microliter or positive leucocyte esterase test with at least two +  + or > 90% polymorphonuclear leucocytes, > 5 polymorphonuclear leucocytes in histology at 400 × magnification, or one positive culture). The anatomical region, incidence of surgical site infection for each subspecialty, and time to revision were also documented.

There was automatic data retrieval from the patient information system (KISIM; Cistec, Zurich, Switzerland) through a search for the according German keywords “infection” and (delayed) “wound healing” in the operating notes. To avoid the risk of misclassification and for quality control, patient charts that were classified as infection were studied only to confirm revision for surgical site infection.

Date are presented as means and standard deviations (SD) as well as absolute numbers and percentages. The percentages for the incidence of surgical site infection for each subspecialty was calculated using the total number of surgeries within each subspecialty. The chi-squared test was used to compare categorical data. A logistic regression analysis adjusts for potential confounders (age, gender, ASA classification, and BMI). The significance level was ≤ 5%. A power calculation was performed and yielded a necessary sample of n ≥ 4′638 (n ≥ 2′319 per group) if the incidence of a surgical site infection was 1% versus 2% in both groups with *P* ≤ 0.05 and a power of 80%. This sample size was met with our data. Stata (IC13.1; StataCorp, College Station, TX, USA) was used for analysis.

## Results

The mean age was 52 (SD 19) years. The cohort included 3′835 (50%) females and 3′905 (50%) males.

The overall incidence of surgical revision for surgical site infection was 0.6 percent (%) (n = 45) in 7′740 included surgical cases. The surgical site infection was superficial in 0.1%, deep in 0.1%, and affected the organ/space in 0.4% according to the criteria by the CDC^14^. The incidence of surgical site infection for each subspecialty was: tumor (1.1% (n = 3, from 275 surgeries)), spine (1.0% (n = 16, from 1′579 surgeries)), hip and knee (0.6% each (n = 8, from 1′243 surgeries; and n = 10, from 1′777 surgeries)), shoulder and foot/ankle (0.3% each (n = 4, from 1′511 surgeries; and n = 4, from 1′335 surgeries)), as well as orthopedic pediatrics (0% (n = 0, from 20 surgeries)) (*P* = 0.09, but *P* = 0.05 if omitting the smallest group (pediatrics)). The mean time to revision was 32 (SD 18) days.

**Figure 2:**
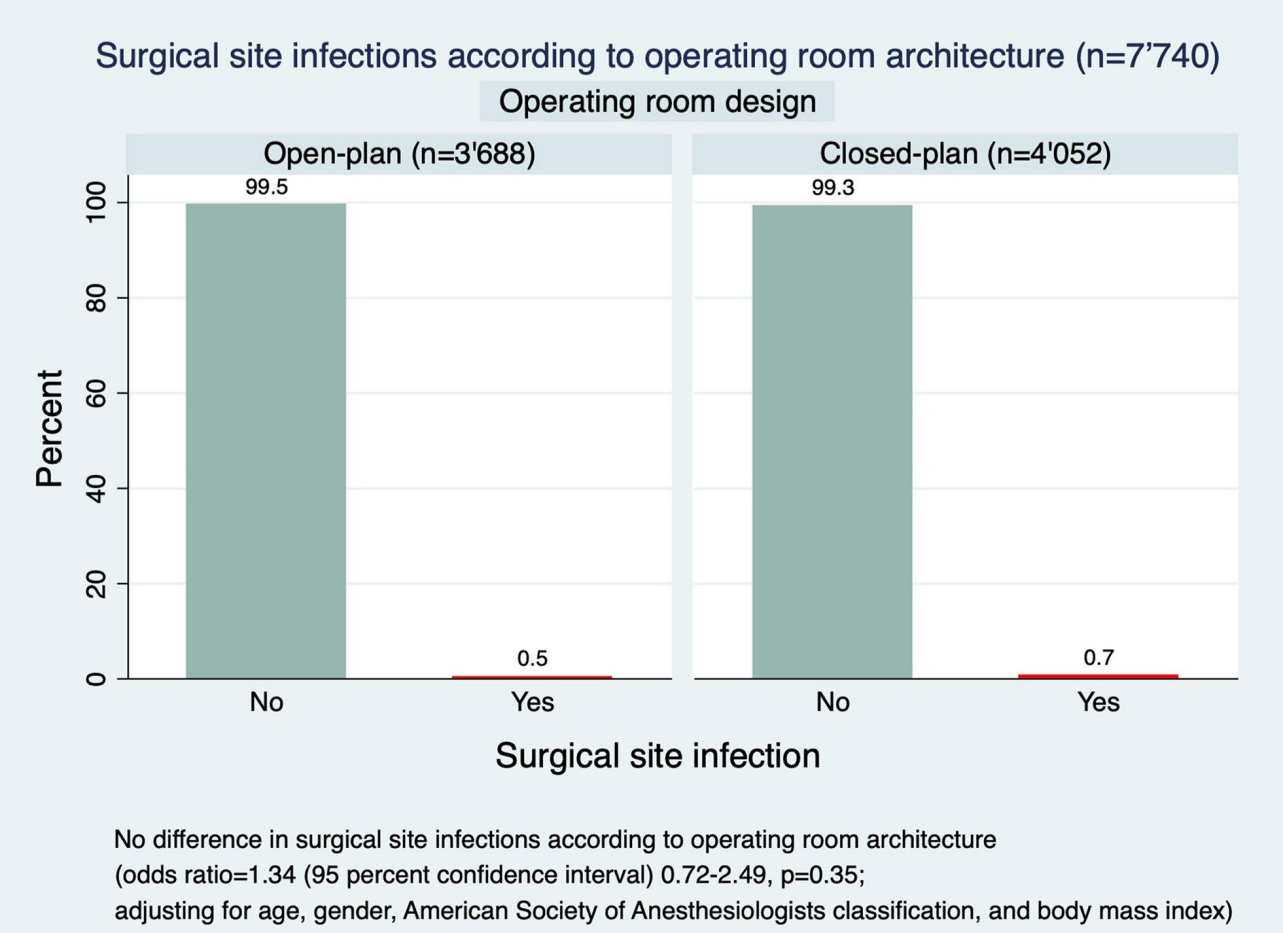
Surgical site infections according to operating room architecture.

There was no difference in incidences of surgical site infection in the open- vs closed-plan operating room architecture (0.5% (n = 18) vs 0.7% (n = 27); adjusted odds ratio (OR) = 1.34 (95% confidence interval (CI) 0.72–2.49, *P* = 0.35)) (Fig. 2 and Tables 1, 2). Age and gender were not a risk factor for surgical site infection (OR = 1.01 (95% CI 0.99–1.03, *P* = 0.54 and OR = 1.18 (95% CI 0.64–2.16, *P* = 0.59, respectively). However, ASA classification and BMI were identified as statistically significant risk factors for the incidence of surgical site infection. The risk of surgical site infection increased by 92% with each increase in one ASA class (OR = 1.92 (95% CI 1.16–3.18], *P* = 0.01). It also increased borderline significantly by 5% with each increase in one kilogram per meter^2^ (kg/m^2^) BMI (OR = 1.05 (95% CI 1.00–1.11], *P* = 0.05).

**Table 1:** Surgical site infection in the open- vs closed-plan operating room architecture (without subspecialties).

Variable	Odds Ratio	(95% CI)	*P*-value*
Operating room architecture	1.34	(0.72–2.49)	0.35
Age	1.01	(0.99–1.03)	0.54
Gender	1.18	(0.64–2.16)	0.59
ASA classification	1.92	(1.16–3.18)	0.01
BMI	1.05	(1.00–1.11)	0.05

*Logistic regression analysis.

% percent, *CI* confidence interval, *ASA* American society of anesthesiologists, *BMI* body mass index.

**Table 2:** Surgical site infection in the open- vs closed-plan operating room architecture with subspecialties.

Variable	Odds Ratio	(95% CI)	*P*-value*
Operating room architecture	1.44	(0.78–2.70)	0.25
Age	1.00	(0.98–1.02)	0.73
Gender	1.17	(0.64–2.15)	0.61
ASA classification	1.78	(1.07–2.96)	0.03
BMI	1.06	(1.01–1.11)	0.03
Subspeciatly†	0.74	(0.60–0.92)	0.01

% percent, *CI* confidence interval, *ASA* American society of anesthesiologists, *BMI* body mass index.

*Logistic regression analysis.

^†^The subspecialty was categorized from the most common to the least common infection rate (as shown in the results section of the second paragraph).

## Discussion

Modifiable and non-modifiable risk factors have been associated with surgical site infection in the past. However the role of the architecture of the operating room was not yet investigated sufficiently, although recent reviews have discussed the importance of gathering evidence-based data on operating room architecture as an integral part of patient care and hospital revenue^6,9^. In this study, the overall incidence of revisions for early infections after a broad spectrum of orthopedic surgeries was low (only 0.6%). As a novel and previously not studied finding, the infection rate was independent of two extreme architectures (open- vs closed-plan) of the operating room. While age and gender were not identifiably risk factors for surgical site infection in this cohort, an increase in ASA classification and, possibly BMI, were identified as independent risk factors for surgical site infection. Although still low, tumor and spine surgeries (around 1% each) showed mildly higher risks for surgical site infection than, for example, shoulder or foot and ankle surgeries (0.3% each).

In our institution, an open-plan operating room architecture was used until 2016 before a newly built closed-plan operating room architecture has been used since 2017. This setup allowed a unique comparison of two extreme architecture concepts. By applying several exclusion criteria, the authors have made an effort to minimize bias and make both groups as otherwise comparable as possible. By further controlling for commonly known potential risk factors for surgical site infection in a logistic regression analysis, results were made more robust. Theoretically, on the one hand, a higher incidence of surgical site infection may be associated with open-plan operating room architecture due to higher door traffic and potential contamination risk^7^. On the other hand, a higher incidence in surgical site infection may be associated with closed-plan operating room architecture due smaller room size with less room around the sterile area and increased likelihood of individuals being in proximity to the sterile surgical field^8^. It remains unknown if these or other aspects may have balanced each other out. When planning new operating rooms in future hospitals, it does not appear to play a major role for the risk of surgical site infections whether the operating room architecture is built in an open- or closed-plan design.

A recent study by Li et al.^12^ investigated 100′815 patients from a large Veterans Administration database and described that surgical site infections are more commonly found in patients with higher ASA classes. While surgical site infections were found in 0.5% of patients with ASA class 1, they were found in 1.0%, 1.9%, 3.4%, and 4.1% for ASA classes 2, 3, 4, and 5. This is in line with our study that showed a 92% increase of risk for surgical site infection with each increase of one ASA class. Furthermore, a review by Xing et al.^15^ identified BMI as an independent risk factor for surgical site infections in spine surgeries. Another study by Si et al.^16^ found that morbidly obese patients (BMI ≥ 40 kg/m^2^) were around 400% more likely to acquire a surgical site infection after total knee arthroplasty. In our study, there was a borderline increase in surgical site infections for each increase in BMI. This is likely due to the fact that surgical site infections are not influenced by obesity in all subspecialties and all types of surgeries, respectively. It has recently been proposed that the distribution of body mass may be a more predictive factor of surgical site infections. Mehta et al.^17^ found that BMI was not associated with infections in spine surgery, but skin to lamina distance and subcutaneous fat thickness were. This could be of interest in future studies.

There are some difficult to avoid limitations that need consideration while interpreting the results. First, we chose revision for surgical site infections as the main outcome. This decision was made since revisions are clearly recorded and documented events whereas wound healing problems are not. This might be considered an underreporting of potentially overseen conservatively treated superficial infections, but if so, such were not relevant enough to undergo surgical revision eventually. Even if this methodological decision would have influenced the overall rate of surgical site infection, it would unlikely have influenced differences between groups. Second, while all factors have remained the same over the years of 2016–2017, the preoperative antibiotic regime was changed from cefuroxime 1.5 g to BMI-adjusted dosage after the first quarter of 2017. This may have introduced some residual confounding, but the authors do not believe there is enough evidence yet to suggest a severe effect on the presented data yet^18–20^. Barbour et al.^19^ studied cefuroxime distributions in the body after intravenous dosage of 1.5 g within one hour of surgical start and found that concentrations in soft tissues were sufficient to prevent gram-positive infections, but may not be sufficient enough to prevent gram-negative infections. Even if this limitation had an effect, it would have reduced the rate of infection in the closed-plan operating room architecture groups and its removal would be in favor of the unexpected results. Third, automatic data retrieval was used from the patient information system by scanning the operating room notes for the keywords “infection*” and (delayed) “wound healing*”. However, the risk of misclassification is very low since all potential infection cases were re-reviewed. It is also unlikely that there are remaining false negatives because the search terms are likely to cover any actual infections. Fourth, there is a remaining risk of a type II error due to limited power because our power calculation assumed a difference in surgical site infection of 1%, but the actual difference was 0.2%. However, since this is the first study about this subject, the sample size is still very large (n = 7′746), and this study could almost be considered a case–control study, we believe that the findings of this study are an important addition to the current literature. Fifth, this study focused only on a few variables as potential causes for infections. These variables were chosen according to previous literature and readily available and reliable data from our database. While the potential infections were confirmed through chart review, it was deemed out of the scope of this study to confirm other data that was not readily available, such as smoking. Future studies may opt to investigate even larger sample sizes and include more variables if possible. Sixth, previous studies have shown that junior surgeons may pose an increased risk (OR = 2.4) of surgical site infections as reported by Hou et al.^21^. In our study, there were a number of different surgeons in each subspecialty. We were not able to control for this potential confounder because we often operate in teams with one senior and one junior surgeon. Each subspecialty has a different number of surgeons, usually ranging around two senior and two junior surgeons. This did not change over the course of the study. Seventh, in addition to the discussed patient-related risk factors for SSIs, there are several other modifiable risk factors that were not investigated in this study, but may be the focus of future studies. As reported by Delmore et al.^22^, perioperative considerations should include the use of clippers (rather than shaving), avoidance of hypothermia, hypovolemia, and hypotension, the use of oxygen, tension-free closure, and surgical drains where necessary.

On a side note, the authors do not feel that the surgical duration varied according to the architecture of the operating rooms, although this could not be studied due to difficulties in obtaining this data. Furthermore, studies may investigate the satisfaction of the surgical and nursing team with different architecture. The open-plan design may enhance communication between different teams and the educational experience, while it also likely has increased background noise and interruptions, potentially limiting concentration causing stress. An interesting study by Bayramzadeh et al.^23^ used an integrative mock-up simulation approach in order to evaluate operating room design prototypes through evidence-based medicine. This could help surgical teams make key decisions about the operating room size and the location of crucial elements, such as the orientation of the operating room table. Stakeholders may benefit from visualizing their workspace and plan accordingly. Finally, the relevance of the findings may extend to low-resources settings, where closed-plan architecture may be associated with additional financial costs.

## Conclusion

The overall incidence of revisions for early infections after a broad spectrum of orthopedic surgeries was relatively low (0.6%) and independent from two extreme forms of operating room architectures. However, biological factors such as increase in ASA scores and possibly BMI were identified as independent risk factors for surgical site infection.
